# Concentration levels of serum 25-Hydroxyvitamin-D and vitamin D deficiency among children and adolescents of India: a descriptive cross-sectional study

**DOI:** 10.1186/s12887-021-02803-z

**Published:** 2021-08-06

**Authors:** Akif Mustafa, Chander Shekhar

**Affiliations:** 1 Research Fellow, International Institute for Population Sciences (IIPS), Mumbai, 400088 India; 2 Department of Fertility studies, International Institute for Population Sciences (IIPS), Mumbai, India

**Keywords:** 25(OH)D, Vitamin D deficiency, 25-hydroxyvitamin D, India, CNNS

## Abstract

**Background:**

Vitamin D is an essential micronutrient for the overall health and well-being of individuals. For strong musculoskeletal and neurological development of human body, vitamin D levels during childhood and adolescence have key importance. This is the first national-level study that analyzes the deficiency and concentration of serum 25-Hydroxyvitamin D [25(OH)D)] among Indian children and adolescents with respect to various demographic and socioeconomic characteristics.

**Methods:**

Data of Comprehensive National Nutrition Survey (CNNS, 2016–18) was utilized for the present study. Vitamin D levels were assessed based on serum 25-hydroxyvitamin D concentration. Prevalence of vitamin D deficiency has been shown for the three age groups: 0–4 years (*n* = 12,764), 5–9 years (*n* = 13,482), 10–19 years (n = 13,065). Vitamin D deficiency was defined as: serum 25(OH)D < 12 ng/mL; and insufficiency as: 12 ng/ml ≤ 25(OH) < 20 ng/ml. 25(OH) D level higher than 20 ng/mL was accepted as adequate. Random slope multilevel logistic regression models were employed to assess the demographic and socioeconomic correlates of vitamin D deficiency.

**Results:**

Mean serum 25(OH)D concentration level was found to be 19.51 ± 8.76, 17.73 ± 7.91, and 17.07 ± 8.16 ng/ml in age group 0–4 years, 5–9 years and 10–19 years respectively. 49.12% of the children aged 0–4 years were having insufficient level of vitamin D. Prevalence of vitamin D deficiency was comparatively higher among female adolescents (76.16%), adolescents living in rural region (67.48), Sikh individuals (0–4 years: 76.28%; 5–9 years: 90.26%; 10–19 years: 89.56%), and adolescents coming from rich households. North-Indian individuals were having substantially higher odds of vitamin D deficiency in all the three age groups.

**Conclusion:**

The present study demonstrated that the prevalence of vitamin D deficiency is considerably high among children and adolescents of India. The study highlights high-risk group which require prompt policy interventions.

**Supplementary Information:**

The online version contains supplementary material available at 10.1186/s12887-021-02803-z.

## Introduction

Vitamin D deficiency is a serious public health problem prevalent worldwide among individuals of all geographic regions, age groups, ethnicities, and races [1]. Earlier, vitamin D was only considered to be important for the prevention of rickets in children, but the recent biomedical and epidemiological research show that vitamin D is essential for the overall health and well-being of individuals. Research shows that insufficient levels of vitamin D are associated with elevated risk of developing numerous skeletal and non-skeletal diseases like colon cancer [2], prostate cancer [3, 4], various other types of cancer [5–9], type-1 diabetes mellitus [10, 11], multiple sclerosis [12], rheumatoid arthritis [13], depression [14], schizophrenia [15], cardiovascular diseases [16], Obesity [17], infectious diseases [18], and various other diseases. Due to diverse and multidimensional impacts on overall health of individuals, vitamin D levels keep an important place in policy formation of a nation or organization.

Vitamin D is a fat-soluble vitamin that promotes mineral homeostasis and absorption of calcium, phosphorous, magnesium, iron, and zinc in the intestine [1]. It plays a pivotal role in maintaining adequate serum calcium and phosphate concentrations which ultimately helps in maintaining normal bone mineralization [19, 20]. Apart from this, it has also been found to be associated with regulation of insulin and glucose secretion [21, 22], and regulation of cardiac and renal functions. Increasing cellular immunity by stimulating production of anti-microbial peptide (cathelicidin) [23], reduction of inflammation, and modulation of cell growth [24] are also influenced by vitamin D.

High prevalence of vitamin D deficiency is being reported all around the world. According to cut off level of 25(OH)D < 20 ng/ml, 24% of the US, 37% of the Canada and 40% of the European population have low levels of vitamin D [25], however, the prevalence varies by age, gender, race, ethnicity, and socioeconomic status. Numerous small scale studies have reported a high prevalence of vitamin D deficiency in Indian subpopulations [26]. In spite of abundant sunshine and being from a tropical region, vitamin D deficiency is significantly high in India. The estimated prevalence of vitamin D deficiency in various population groups across India varies from 34 to 94% [27]; however, we did not found any large scale study which has provided nationally representative estimates of vitamin D deficiency.

Major skeletal and neurological development of the human body takes place during childhood and adolescence. For vigorous physical and mental development, vitamin D levels during childhood and adolescence have key importance. High prevalence of vitamin D deficiency has been reported among adolescents and children worldwide [28–30]. A few small scale studies on vitamin D deficiency among children and adolescents have been conducted in India, but none of them was nationally representative [19, 31]. Hence the present study makes an effort to comprehensively analyze the deficiency and levels of vitamin D among Indian children and adolescents with respect to various demographic and socioeconomic characteristics using nationally representative data. The study will also explore the demographic and socioeconomic correlates of vitamin D deficiency among adolescents using robust statistical analyses.

## Methodology

### Data

The present study utilizes the data of the Comprehensive National Nutrition Survey (CNNS, 2016–18). CNNS provides robust data on nutritional status, anthropometric indicators, food intake, and micronutrient levels of children and adolescents aged 0 to 19 years. The survey was the largest micronutrient survey globally. The CNNS was conducted under the stewardship of Ministry of Health and Family Welfare (MohFW), Government of India; technical support was provided by UNICEF, over field coordination training, and data analysis was conducted by Population Council. The survey adopted a multi-stage sampling design to collect data from 30 states of India. The data collection was done for three target groups: preschoolers (0–4 years), school-age children (5–9 years), and adolescents (10–19 years). The total planned national-level sample size was 122,100 children and adolescents from 2035 primary sampling units. For each of the three age groups, the planned sample size was 40,700 individuals for household survey and anthropometric measurements; and for biological samples, the sample size was 20,350 individuals from each group. The overall response rate for individual interviews was 95%; on the other hand, the response rates for biological sample collection were comparatively low: 63–64% in the three age groups. Detailed description of household selection, questionnaire, data collection, instruments used, and data handling can be accessed from the CNNS (2016–18) report [32].

### Blood collection and serum 25(OH)D estimation

Parents and subjects were instructed regarding overnight fasting (8–10 h) 1 day before the sample collection. The venous blood samples were collected by trained phlebotomists. The status of fasting (yes/no) was also asked before the blood sample collection. The blood samples were transported to the nearest collection center in cold bags. There, the serum was separated and divided into aliquots within 6 h of sample collection. The aliquots were then stored at − 20 °C until the analysis. The 25(OH)D concentration was measured with an antibody competitive immunoassay using the chemiluminescence (Siemens Centaur) method. The biochemical analysis was performed by SRL labs. With standard internal and external quality assurance procedures, extensive quality control measures were taken during the data collection [32].

### Sample size

Although the planned sample size was 20,350 individuals from each age group, but due to missing values, non-response, insufficient quality, and invalid observations, the final sample size of the study was reduced. For the 0–4 age group, the sample size was 12,764, for 5–9 age group 13,482, and for 10–19 age group, the sample size was 13,065. For the national representation of the estimates, sampling weights were utilized, which were provided in the CNNS dataset.

### Outcome variable

Serum 25-hydroxyvitamin D [25(OH)D] is considered as the most reliable indicator of vitamin D levels in the human body [1]. The serum 25(OH)D concentration level reflects not only cutaneous synthesis of vitamin D but also dietary absorption of vitamin D. In the present study, therefore, 25(OH)D concentration level was taken as the principal outcome variable. There are two established cut-offs of 25(OH)D concentration for indication of vitamin D deficiency: < 12 ng/ml defined by National Academy of Medicine, USA and < 20 ng/ml defined by Endocrine Society, USA. However, most of the researchers follow criteria suggested by ‘Munns et al.’ [33]. According to their criteria, vitamin D deficiency is defined as serum 25(OH)D level of < 12 ng/mL and insufficiency as 25(OH) D level between 12 and 20 ng/mL. 25(OH) D level higher than 20 ng/mL was accepted as adequate. We have also used the criteria suggested by ‘Munns et al.’ to define vitamin D deficiency and insufficiency in this study [33]. For regression analysis a new binary variable was defined as 1 = “deficient” (< 12 ng/ml) and 0 = “Not deficient” (≥ 12 ng/ml).

### Predictor variables

All the available demographic and socio-economic variables were included in the regression analysis as predictor variables. The variables are as follows: Age of the individual (in months), Gender (Male, Female), Place of residence (Urban, Rural), Wealth Index (Poorest, Poor, Middle, Rich, Richest), Religion (Hindu, Muslim, Christian, Sikh, Other), Caste (Scheduled Caste(SC), Scheduled Tribe (ST), Other Backward Class (OBC), and Others), Mothers Education Level (no education, < 5 years completed, 5–8 years completed, 9–11 years completed, ≥ 12 years completed), Region (North, Central, East, West, South, North-East). The results were controlled for BMI, cholesterol and seasonal variation. The results of the controlled variables are presented in supplementary file. (Note: The wealth index was calculated on the basis of possession of common household items and facilities as described in National Family Health Survey - 4 [34].)

### Statistical analyses

The association between vitamin D deficiency and demographic & socioeconomic variables was assessed using two-level random slope logistic regression models. As the data was hierarchical in nature and observations were clustered within PSUs, there were high chances that the observations were correlated within PSUs. So a simple logistic regression might suffer from intra-cluster correlation and provide less reliable estimates. This issue can be solved using a multilevel model that will not only control for intra-cluster correlation but also community variables like development level, political profile, climatic conditions, etc. PSUs were set as level-2 in the multilevel logistic regression model. Variance inflation factors were calculated to detect any kind of multi-collinearity in the model. No evidence of multi-collinearity was found in the analysis. All the statistical analyses were performed on STATA 16 [35].

## Results

Table 1 is showing the age group wise prevalence of vitamin D deficiency, insufficiency and mean serum 25(OH)D. The prevalence of vitamin deficiency was 14, 19 and 24% in the age groups 0–4, 5–9 and 10–19 respectfully. 68% of the adolescents were having insufficient levels of vitamin D. Mean serum 25(OH)D level was highest in 0–4 years age group and lowest in 10–19 years age group.

**Table 1:** Prevalence of vitamin D deficiency and insufficiency (in percentage) and average serum 25(OH)D concentration level among children and adolescents of Indian

Indicator	Age Group		
	0–4	5–9	10–19
**Vitamin D deficiency (25(OH)D < 12 ng/mL)**	14.44	18.89	23.91
**Vitamin D insufficiency (25(OH)D < 20 ng/ml)**	49.12	56.06	68.45
**Mean serum 25(OH)D level**	19.51 ± 8.76	17.73 ± 7.91	17.07 ± 8.16

Table 2 illustrates average concentration levels of serum 25(OH)D by background characteristics among Indian children and adolescents. In all the three age groups, the 25(OH)D levels were higher for males than females. The levels of 25(OH)D were lower among urban individuals compared to rural individuals. The 25(OH)D levels were substantially low among Sikh individuals in all the three age groups. Children and adolescents of the northern region of the country were having significantly low levels of 25(OH)D; on the other hand, individuals of the southern region were having comparatively high levels of 25(OH)D.

**Table 2:** Concentration levels of serum 25(OH)D among Indian children and adolescents

	25(OH)D, ng/mL; mean (SD)		
		Age group	
	0–4	5–9	10–19
**Gender**			
Male	19.62 (8.94)	18.30 (8.03)	19.06 (8.27)
Female	19.39 (8.56)	17.08 (7.73)	14.96 (7.50)
**Place of Residence**			
Urban	20.64 (8.76)	18.78 (7.78)	18.03 (8.15)
Rural	18.04 (8.55)	16.34 (7.87)	15.80 (8.01)
**Wealth Index**			
Poorest	21.22 (8.28)	19.97 (7.04)	19.38 (7.84)
Poor	20.78 (8.41)	19.68 (7.83)	18.80 (8.05)
Middle	20.51 (8.65)	18.73 (7.70)	18.13 (8.02)
Rich	19.19 (8.40)	17.56 (7.88)	16.44 (8.10)
Richest	18.22 (9.18)	16.13 (7.91)	15.64 (8.09)
**Religion**			
Hindu	19.73 (8.87)	17.85 (7.87)	17.28 (8.15)
Muslim	19.10 (8.95)	17.64 (8.23)	15.84 (8.07)
Christian	19.76 (7.79)	18.33 (7.57)	18.23 (8.20)
Sikh	14.57 (8.56)	11.62 (7.13)	11.95 (7.58)
Other	19.20 (8.05)	17.76 (7.50)	16.89 (7.26)
**Caste**			
SC	19.63 (9.09)	17.81 (8.05)	16.93 (8.18)
ST	20.55 (8.01)	18.83 (7.39)	18.47 (7.99)
OBC	20.55 (8.01)	18.19 (8.09)	17.59 (8.29)
Others	19.78 (9.11)	16.09 (7.81)	15.39 (8.01)
**Mothers Education**			
No education	20.30 (8.84)	18.69 (7.88)	18.03 (8.35)
< 5 years completed	20.34 (8.27)	18.27 (7.32)	17.69 (7.73)
5–8 years completed	19.40 (8.47)	17.63 (7.78)	16.72 (7.97)
9–11 years completed	19.71 (8.92)	17.81 (8.21)	16.47 (7.94)
≥ 12 years completed	18.59 (8.87)	16.18 (7.82)	15.09 (8.20)
**Zone**			
North	16.42 (9.11)	14.18 (7.51)	14.16 (7.78)
Central	19.83 (7.12)	18.57 (7.03)	17.91 (7.64)
East	19.91 (7.86)	17.67 (6.89)	16.61 (7.29)
West	20.07 (9.68)	18.86 (8.75)	18.39 (8.82)
South	22.23 (9.21)	20.83 (8.36)	19.90 (8.52)
North-East	19.29 (8.34)	17.98 (7.51)	17.26 (7.92)

**Fig. 1:**
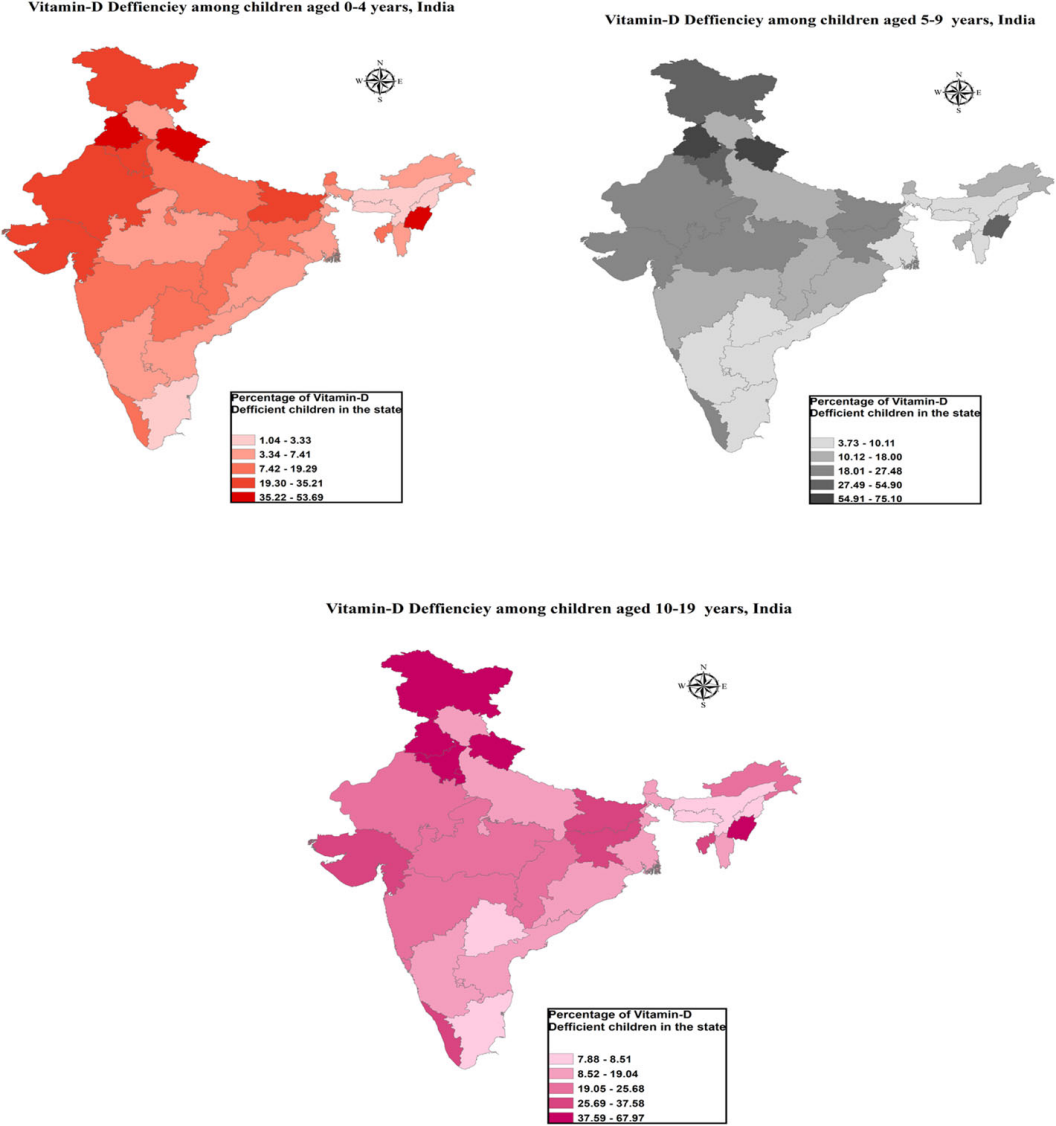
Choropleth maps of vitamin deficiency among children and adolescents in India

Figure 1 is showing the choropleth maps of vitamin D deficiency in India according to cut-off level of serum 25(OH)D < 12 ng/mL. From the graphs, it can be clearly visualized that the prevalence of vitamin D deficiency is comparatively low in southern and north-eastern regions of the country and very high in northern and western regions of the country.

The prevalence of vitamin D deficiency with respect to demographic and socioeconomic characteristics is presented in Table 3. In the age group 0–4 and 5–9, there was not much difference in the prevalence of deficiency between males and females, however, in 10–19 years age group the prevalence was very high among female adolescents compared to male adolescents. Vitamin D deficiency was more prevalent among children and adolescents of rich households compared to adolescents of poor households. The prevalence was substantially high among Sikh children; 89.59% of the Sikh children of age group 10–19 years were having insufficient levels of vitamin D.

**Table 3:** Prevalence of vitamin D deficiency and insufficiency (in %) with respect to demographic and socioeconomic characteristics

	Age Group					
	0–4		5–9		10–19	
	Deficiency (< 12 ng/mL)	Insufficiency (< 20 ng/ml)	Deficiency (< 12 ng/mL)	Insufficiency (< 20 ng/ml)	Deficiency (< 12 ng/mL)	Insufficiency (< 20 ng/ml)
**Gender**						
Male	14.05	47.78	18.02	53.80	14.82	49.59
Female	14.87	50.61	19.81	58.47	35.47	76.16
**Place of Residence**						
Urban	21.68	59.3	28.63	52.90	35.35	60.21
Rural	12.29	46.1	16.19	67.48	21.94	71.02
**Wealth Index**						
Poorest	14.72	44.42	14.26	48.93	19.82	57.55
Poor	11	43.28	13.98	48.68	18.92	56.19
Middle	10.48	46.47	16.12	55.07	21.12	59.50
Rich	15.91	52.45	21	60.18	29.49	68.17
Richest	20.78	58.50	29.74	67.73	35.91	72.04
**Religion**						
Hindu	13.75	48.45	18.68	55.76	23.23	61.65
Muslim	14.51	51.26	14.99	53.92	31.31	67.00
Christian	9.81	41.54	20.68	59.46	17.39	55.28
Sikh	54.14	76.28	70.9	90.26	68.91	89.56
Other	19.49	50.68	17.75	56.91	24.2	56.16
**Caste**						
SC	20.61	53.21	20.61	59.32	27.6	62.39
ST	15.8	39.66	15.8	51.28	15.13	58.32
OBC	18.66	51.42	18.66	54.66	23.6	61.84
Others	22.13	51.42	22.13	54.66	30.28	66.09
**Mothers Education**						
No education	13.85	45.47	17.01	50.00	22.36	58.35
< 5 years completed	10.23	42.12	14.88	61.43	25.42	65.32
5–8 years completed	15.02	51.83	20.4	59.48	26.56	65.78
9–11 years completed	13.94	48.57	18.42	56.91	27.61	67.85
≥ 12 years completed	16.6	53.62	25.88	63.94	34.69	72.12
**Zone**						
North	35.08	65.12	41.69	71.42	43.93	69.99
Central	12.26	46.04	15.18	51.48	19.98	58.32
East	14.06	51.99	18.82	60.62	27.25	69.89
West	18.39	50.39	20.71	53.52	26.23	59.27
South	5.52	39.62	9.01	48.37	14.57	55.84
North-East	8.99	40.85	10.83	40.88	13.95	45.71

*Note: Numbers are showing percentage of vitamin D deficiency*

The results of multilevel logistic regressions are presented in Table 4. Compared to males, females were having 1.54 (OR: 1.54; 95% CI: 1.39–1.71) and 3.8 (OR: 3.8; 95% CI: 3.42–4.22) times higher odds of being vitamin deficient in 5–9 and 10–19 years age group respectively. Results show that in the three age groups the odds of being vitamin D deficient are significantly higher among urban individuals compared to rural individuals. Wealth Index did not found to be strongly associated with vitamin D deficiency among children of 0–4 age group, however, in 5–9 and 10–19 years, age group the individuals of the richest households were having 56% (OR: 1.56; 95% CI: 1.14–2.14) and 65% (OR: 1.65; 95% CI: 1.25–2.18) higher odds of being vitamin D deficient respectively compared to individuals of the poorest households. Children of Sikh family were at high risk of vitamin D deficiency, the odds of having vitamin D deficiency were 2.1 (OR: 2.1; 95% CI: 1.39–3.19) times higher among 10–19 year old adolescents of Sikh family compared to adolescents of Hindu family. Children and adolescents of ST households were having significantly lower odds of being vitamin D deficient compared to other caste groups. Geographical region of the country was strongly associated with vitamin D deficiency among children and adolescents. As the mean 25(OH)D levels were highest in southern region so we took south region of the country as the base category. The risk of vitamin D deficiency was highest in northern region, a 5–9 year old individual living in northern region of the country was 10.4 (OR: 10.44; 95% CI: 7.76–14.05) times more likely to be vitamin D deficient compared to a 5–9 year old individual living in southern region. In 4–9 and 5–9 year age group, age (in months) of the child was positively associated with odds of vitamin D deficiency, in both the age groups with 1 month increase in age was associated with 1% (OR: 1.01; 95% CI: 1.005–1.015) increase in odds of vitamin D deficiency; however, in 10–19 years age group the association was other way round i.e. with 1 month increase in age there was decrement of 1% (OR: 0.997; 95% CI: 0.9954–0.9983) in odds of being vitamin D deficient. The intra-class correlation was statistically significant in all the three regression models.

**Table 4:** Results of multilevel logistic regression analysis assessing the odds of vitamin D deficiency among children and adolescents of India, CNNS 2016–18

	Age Group					
	0–4		5–9		10–19	
	OR	95% CI	OR	95% CI	OR	95% CI
**Gender**						
Male	1	1	1	1	1	1
Female	1.06 ns	0.94–1.20	1.54***	1.39–1.71	3.80***	3.42–4.22
**Place of Residence**						
Rural						
Urban	2.1***	1.70–2.56	1.96***	1.63–2.35	1.81***	1.53–2.14
**Wealth Index**						
Poorest	1	1	1	1	1	1
Poor	0.91 ns	0.67–1.26	1.10 ns	0.82–1.48	1.06 ns	0.81–1.39
Middle	0.74*	0.54–1.04	1.34**	1.002–1.79	1.16 ns	0.89–1.51
Rich	0.89 ns	0.63–1.24	1.43**	1.06–1.92	1.60***	1.23–2.08
Richest	0.98 ns	0.69–1.40	1.56***	1.14–2.14	1.65***	1.25–2.18
**Religion**						
Hindu	1	1	1	1	1	1
Muslim	1.04 ns	0.81–1.32	0.94 ns	0.76–1.16	1.59***	1.31–1.91
Christian	0.90 ns	0.64–1.27	1.44**	1.10–1.90	0.87 ns	0.66–1.15
Sikh	1.72**	1.11–2.68	2.04***	1.34–3.12	2.10***	1.39–3.19
Other	0.98 ns	0.68–1.41	1.04 ns	0.73–1.48	0.80 ns	0.56–1.31
**Caste**						
Others	1	1	1	1	1	1
SC	0.97 ns	0.79–1.18	0.91 ns	0.76–1.08	1.05 ns	0.88–1.24
ST	0.70**	0.52–0.93	0.65***	0.51–0.82	0.67***	0.53–0.84
OBC	1.10 ns	0.91–1.32	1.01 ns	0.86–1.17	1.02 ns	0.87–1.19
**Mothers Age at childbirth**						
< 18 years	1	1	1	1	1	1
18–25 years	1.44 ns	0.89–0.34	1.20 ns	0.89–1.60	0.98 ns	0.81–1.18
26–34 years	1.41 ns	0.87–2.30	1.36*	1.01–1.83	0.98 ns	0.81–1.20
35+	1.63*	0.94–2.81	1.52*	1.06–2.19	1.08 ns	0.82–1.42
Mother died	1.50 ns	0.43–0.51	1.73 ns	0.82–3.67	0.94 ns	0.60–1.47
**Mothers Education**						
No education	1	1	1	1	1	1
< 5 years completed	0.87 ns	0.62–1.23	1.09 ns	0.86–1.37	1.08 ns	0.88–1.34
5–8 years completed	1.05 ns	0.85–1.29	1.06 ns	0.90–1.25	1.18**	1.02–1.37
9–11 years completed	1.06 ns	0.84–1.34	1.18*	0.98–1.42	1.29**	1.10–1.52
≥ 12 years completed	1.07 ns	0.85–1.36	1.36**	1.16–1.66	1.65***	1.36–2.01
**Region**						
South	1	1	1	1	1	1
North	9.85***	6.99–13.89	10.44***	7.76–14.05	8.14***	6.18–10.71
Central	2.14***	1.44–3.17	2.73***	1.90–3.91	2.79***	2.01–3.88
East	3.29***	2.33–4.65	3.58***	2.61–4.91	3.80***	2.85–5.07
West	4.32***	3.00–6.20	3.31***	2.34–4.69	2.71***	1.96–3.74
North-East	3.32***	2.30–4.80	2.34***	1.69–3.24	3.31***	2.45–4.47
**Age in months**	1.01***	1.005–1.015	1.01***	1.007–1.014	0.997***	0.9954–0.9983
Random effect (standard error)	2.03 (0.169)		1.96 (0.137)		1.63 (0.118)	
ICC (standard error)	0.38 (0.019)		0.37 (0.016)		0.33 (0.016)	

Note: ***: p-value < 0.001; **: p-value < 0.05; *: p-value < 0.1; ***ns****: not significant; ICC: Intra-cluster correlation*

## Discussion

In the present study, we have tried to assess the serum 25(OH)D profile and vitamin D deficiency of Indian children and adolescents. Up to the best of our knowledge, this is the first national-level study of vitamin D deficiency among children and adolescents in India to date. The study finds a significantly high prevalence of vitamin D deficiency in all the three age groups; however, the prevalence was highly variable between the demographic and socioeconomic categories.

Ultraviolet B (UVB) (290-320 nm) radiation from sunlight plays the most crucial role in the production of vitamin D_3_(cholecalciferol) in the human body. Cutaneous exposure of UVB is channelized through numerous Individual factors such as skin pigmentation (melanin concentration in the skin), clothing behavior, use of sunscreen creams, sun avoidant lifestyle, etc. In the present study also, variation in serum 25(OH)D concentration level might be explained by extent of sunlight exposure among individuals of various demographic and socioeconomic categories. Like some European and middle-eastern countries, there is no policy of vitamin D fortification of food products in India; hence sunlight exposure and skin synthesis is the main source of vitamin D production in the body among the Indians. The present study shows that the prevalence of vitamin D deficiency was higher in rural areas compared to urban areas; this can be simply explained by the fact that people living in rural areas are more exposed to sunlight compared to people living in urban areas. Most of the people in rural areas do agricultural work and their children also help them in the field, exposing them to sunlight for a significant amount of time; on the other hand, people in urban areas generally try to keep their children away from sunlight. Similarly, we also found that serum 25(OH)D concentration levels are considerably high among poor people compared to rich people; again the main factor is lifestyle: generally, rich people in India have sun avoidant lifestyle, on the other hand, poor people’s lifestyle makes them exposed to sunlight which ultimately leads to higher concentrations of serum 25(OH)D. similar kinds of results were found in a study conducted on healthy school girls of Delhi [31].

Among adolescents, serum 25(OH)D concentration was substantially higher among males compared to females. Generally, males are more involved in outdoor activities than females in India, which might be one of the reasons of elevated serum 25(OH)D concentration among males. Apart from that, use of sunscreen products and sun avoidant behavior is more common among females than males [36]. Similar results are found in numerous previous studies [36–38]. However, in some other studies, the results were other way round, i.e. serum 25(OH)D levels were higher among females compared to males [39, 40].

We also observed that vitamin D deficiency was alarmingly high among Sikh children and adolescents. From the literature, we did not find any convincing explanation of low vitamin D levels among Sikh individuals. Further research on causes and correlates of low serum 25(OH)D levels among Sikh individuals is required.

Results of the present study reveal that the concentration of serum 25(OH)D was higher among individuals of the southern region of the country and substantially low among individuals of the northern region of the country, even when urbanization is higher in southern India compared to northern India. There can be numerous reasons for this, but the most convincing reason we found was ‘Ultraviolet Index’ (UV Index); UV index tells us the expected amount of UV radiation that will reach the earth’s surface at the time when the sun is highest in the sky. The amount of UV radiation reaching the surface is affected by elevation of the sun in the sky, the amount of ozone in the stratosphere, and the amount of cloud cover. The UV index ranges from 0 to 13: higher the UV index higher the amount of UV radiation reaching the surface. The UV index in northern region of India ranges from 3 to 7, on the other hand, UV index in southern region of the country varies from 6 to 13 [41] i.e. the amount of UV radiation exposure is much higher in southern India compared to northern India. This might be one of the major reasons of higher vitamin D levels among southern India Individuals. Another point is that northern India comprises of states like Himachal Pradesh and Jammu Kashmir which have hilly landscapes and cold weather i.e. people wear full clothes to keep them warm resulting in less exposure to sunlight.

Though exposure to sunlight is indeed very important for the production of vitamin D in the body but exposure to UV radiation has some serious side effects also. It is a well-established fact that chronic exposure to Ultraviolet-B (UV-B) radiation can increase the risk of nonmelanoma skin cancer (NMSC) in humans [42]. UV-B radiation of 290 to 320 wavelength present in the solar spectrum can cause erythema, burns, damage in DNA, and skin cancer [42]. That is why direct exposure of sunlight to skin is not recommended by doctors and scientists, especially during peak hours (mid-day); however, early morning and late evening sunlight is considered safe, healthy, and beneficial [43, 44].

From the literature, there are a few recommendations to deal with vitamin D deficiency among children and adolescents in India:

- **Fortification of food products with vitamin D:** Fortification is a well-known strategy to deal with deficiency of any micronutrient in the population [45]. The government can introduce protocols for mandatory or voluntary fortification of selected food products. Fortification of food staple by government mandate is the most effective method of increasing dietary intake of a particular micronutrient in the population [45]. Currently, there is mandatory vitamin D fortification programs for specific food products are running in Canada and United States [46].
- **Vitamin D supplementation:** Vitamin D supplementation to the target groups in the form of tablets, capsules, drops can be a potential way to elevate vitamin D levels among the high-risk groups. Supplementation of 400 IU/day to breast-feeding infants is extensively recommended and practiced in numerous first world countries like Canada and United States [24, 33]. However, the WHO does not recommend routine supplementation of vitamin D to children.
- **Health education regarding sunlight:** Government can promote awareness regarding the benefits of sunlight through mass media like radio, television and social media. Previously Indian government has run various health awareness programs pertaining to polio, HIV, tuberculosis, etc. Similarly, a well-structured program focusing on key points like the best time for sunlight exposure, detrimental effects of vitamin D deficiency, and the importance of vitamin D in the body can help in increasing awareness in the population.
- A well-structured school level program focusing on outdoor physical activities for a specific period of time during school hours can be instrumental in dealing with vitamin D deficiency among children and adolescents.

### Limitations

The unavailability of data on important aspects related to vitamin D was one of the major limitations of the study. One of the most important factors that affect vitamin D levels in the body is the amount of time spent in sunlight, and CNNS does not provide information on this, that is why we could not control for it in regression analysis, which puts the results on risk of omitted variable bias.

## Conclusion

The present study portrays the national level profile of serum 25(OH)D among children and adolescents of India. In conclusion, it can be said that there is significant level of vitamin D deficiency among children and adolescents in India, and certain demographic and socioeconomic groups are at a very high risk of complications related to vitamin D deficiencies. Exposure to sunlight has a very important role in channelizing vitamin D levels among Indian children and adolescents. We believe that the results of the present study have important policy implications.

## Supplementary Information

**Additional file 1.** Supplementary Table S1: Results of logistic regression assessing odds of vitamin D deficiency in categories of the controlled variables.

## Data Availability

The dataset utilized in the present study can be accessed from Population Council (India) on reasonable request basis.

## Abbreviations

CNNS: Comprehensive National Nutrition Survey
25(OH)D: 25-Hydroxyvitamin-D
UV-B: Ultra Violet – B
IU/day: International Unit per day
BMI: Body Mass Index
ng/ml: Nanograms per milliliter
nmol/liter: Nanomoles per liter
SC: Scheduled Caste
ST: Scheduled Tribe
OBC: Other Backward Category
