# Supplementary material for: Concentration levels of serum 25-Hydroxyvitamin-D and vitamin D deficiency among children and adolescents of India: a descriptive cross-sectional study
Source: BMC Pediatr. 2021 Aug 6;21:334. doi: 10.1186/s12887-021-02803-z (PMC8344146; doi:10.1186/s12887-021-02803-z)
Supplement: Supplementary file 1 — Additional file 1. Supplementary Table S1: Results of logistic regression assessing odds of vitamin D deficiency in categories of the controlled variables. [file 12887_2021_2803_MOESM1_ESM.docx]

| **Supplementary Table S1**: Results of logistic regression assessing odds of vitamin D deficiency in categories of the controlled variables | | | | | | | |
| --- | --- | --- | --- | --- | --- | --- | --- |
|  |  | **Age Group** | | | | | |
|  |  | **0 - 4** | | **5 - 9** | | **10 - 19** | |
|  |  | OR | 95% CI | OR | 95% CI | OR | 95% CI |
| **BMI** | |  |  |  |  |  |  |
|  | quantile-1 | 1 | - | 1 | - | 1 | - |
|  | quantile-2 | 1.04ns | 0.87 - 1.24 | 1.16* | 1.00 - 1.34 | 1.17** | 1.01 - 1.35 |
|  | quantile-3 | 1.36*** | 1.13 - 1.63 | 1.20** | 1.03 - 1.40 | 1.22** | 1.04 - 1.43 |
|  | quantile-4 | 1.34*** | 1.10 - 1.64 | 1.50*** | 1.25 - 1.79 | 1.50*** | 1.27 - 1.77 |
| **Cholesterol** | |  |  |  |  |  |  |
|  | quantile-1 |  |  | 1 | - | 1 | - |
|  | quantile-2 |  |  | 1.10ns | 0.94 - 1.28 | 1.12ns | 0.97 - 1.30 |
|  | quantile-3 |  |  | 1.10ns | 0.94 - 1.28 | 1.28*** | 1.10 - 1.49 |
|  | quantile-4 |  |  | 1.15* | 0.98 - 1.35 | 1.41*** | 1.21 - 1.65 |
| **Month** | |  |  |  |  |  |  |
|  | Jan | 1 | - | 1 | - | 1 | - |
|  | Feb | 1.11ns | 0.74 - 1.70 | 1.32ns | 0.89 - 1.96 | 1.04ns | 0.72 - 1.52 |
|  | March | 0.74ns | 0.49 - 1.12 | 0.91ns | 0.63 - 1.34 | 0.81ns | 0.57 - 1.16 |
|  | April | 0.66ns | 0.45 - 1.00 | 0.68* | 0.47 - 1.00 | 0.58*** | 0.41 - 0.82 |
|  | may | 0.28*** | 0.18 - 0.44 | 0.21*** | 0.14 - 0.32 | 0.30*** | 0.21 - 0.43 |
|  | June | 0.37*** | 0.25 - 0.57 | 0.34*** | 0.24 - 0.51 | 0.27*** | 0.20 - 0.39 |
|  | July | 0.26*** | 0.18 - 0.40 | 0.25*** | 0.18 - 0.36 | 0.22*** | 0.17 - 0.31 |
|  | August | 0.24*** | 0.16 - 0.36 | 0.24*** | 0.17 - 0.36 | 0.23*** | 0.17 - 0.33 |
|  | Sept. | 0.28*** | 0.19 - 0.42 | 0.27*** | 0.19 - 0.39 | 0.20*** | 0.15 - 0.28 |
|  | October | 0.37*** | 0.22 - 0.63 | 0.34*** | 0.22 - 0.52 | 0.24*** | 0.17 - 0.36 |
|  | Nov. | 1.07ns | 0.70 - 1.66 | 0.99ns | 0.68 - 1.47 | 0.65** | 0.46 - 0.94 |
|  | December | 0.73ns | 0.51 - 1.06 | 1.03ns | 0.74 - 1.44 | 0.71ns | 0.52 - 0.97 |
| Note: ***: p-value < 0.001; **: p-value < 0.05; *: p-value <0.1; ns: not significant; ICC: Intra-cluster correlation | | | | | | | |
| Cholesterol levels were not measured for the age group 0-4 years | | | | | | | |
